# Supplementary material for: Effect of harvest on the agronomic, mineral and antioxidant profile of three oregano species (Origanum onites L., Origanum vulgare L. ssp. hirtum, and Origanum acutidens (Hand.-Mazz.) Ietswaart)
Source: PeerJ. 2025 Oct 21;13:e20223. doi: 10.7717/peerj.20223 (PMC12551659; doi:10.7717/peerj.20223)
Supplement: Supplemental Information 3 [file peerj-13-20223-s003.docx]

Antioxidant v1

Excel pages

DPPH 1.T 🡪 DPPH 1.Repicate

DPPH 1.T 🡪 DPPH 2.Repicate

DPPH 1.T 🡪 DPPH 3.Repicate

DPPH 1.T Page

9 Numaralı Numune 🡪 Sample 9

10 Numaralı Numune 🡪 Sample 10

11 Numaralı Numune 🡪 Sample 11

12 Numaralı Numune 🡪 Sample 12

13 Numaralı Numune 🡪 Sample 13

14 Numaralı Numune 🡪 Sample 14

15 Numaralı Numune 🡪 Sample 15

16 Numaralı Numune 🡪 Sample 16

17 Numaralı Numune 🡪 Sample 17

Kontrol –> Control

DPPH 2.T Page

9 Numaralı Numune 🡪 Sample 9

10 Numaralı Numune 🡪 Sample 10

11 Numaralı Numune 🡪 Sample 11

12 Numaralı Numune 🡪 Sample 12

13 Numaralı Numune 🡪 Sample 13

14 Numaralı Numune 🡪 Sample 14

15 Numaralı Numune 🡪 Sample 15

16 Numaralı Numune 🡪 Sample 16

17 Numaralı Numune 🡪 Sample 17

Kontrol –> Control

DPPH 3.T Page

9 Numaralı Numune 🡪 Sample 9

10 Numaralı Numune 🡪 Sample 10

11 Numaralı Numune 🡪 Sample 11

12 Numaralı Numune 🡪 Sample 12

13 Numaralı Numune 🡪 Sample 13

14 Numaralı Numune 🡪 Sample 14

15 Numaralı Numune 🡪 Sample 15

16 Numaralı Numune 🡪 Sample 16

17 Numaralı Numune 🡪 Sample 17

Kontrol –> Control

FRAP.xlsx

Boş 🡪 Blank

1.Grup 🡪 1. Group

2.Grup 🡪 2. Group

3.Grup 🡪 3. Group

1T 🡪 1 Replicate (R) 🡪 1R

2T 🡪 2R

3T 🡪 3R

Ort. –> Mean

ICP-MS.xlsx

1. Tekrar 🡪 1. Replicate

2. Tekrar 🡪 2. Replicate

3. Tekrar 🡪 2. Replicate

Ortalama –> Mean

TFC-1.xlsx

1T 🡪 1 Replicate (R) 🡪 1R

2T 🡪 2R

3T 🡪 3R

4T 🡪 4R

Ort. –> Mean

**Quercetin page**

Kontrol –> Control

ORT 🡪 Mean

TPC v1.xlsx

Gallik Asid –> Gallic acids

1T 🡪 1 Replicate (R) 🡪 1R

2T 🡪 2R

3T 🡪 3R

4T 🡪 4R

Ort. –> Mean

**Phenolic Sample Page**

K-11'den itibaren 🡪 Control after sample 11

1T 🡪 1 Replicate (R) 🡪 1R

2T 🡪 2R

3T 🡪 3R

4T 🡪 4R

ABTS.xlsx

1T 🡪 1 Replicate (R) 🡪 1R

2T 🡪 2R

3T 🡪 3R

4T 🡪 4R

Ort. –> Mean

Standart –> Standard or Reference
